# Where *are* biomedical research plain‐language summaries?

**DOI:** 10.1002/hsr2.175

**Published:** 2020-08-10

**Authors:** Hannah FitzGibbon, Karen King, Claudia Piano, Carol Wilk, Mary Gaskarth

**Affiliations:** ^1^ CMC Affinity and CMC Connect McCann Health Medical Communications Macclesfield UK; ^2^ CMC Affinity McCann Health Medical Communications Glasgow UK; ^3^ Formerly of CMC Connect McCann Health Medical Communications Philadelphia Pennsylvania; ^4^ Formerly of CMC Affinity McCann Health Medical Communications Hackensack New Jersey; ^5^ CMC Affinity McCann Health Medical Communications Macclesfield UK

**Keywords:** biomedical research, communication, lay summaries, patient summaries, plain English summaries, plain‐language summaries

## Abstract

**Background and Aims:**

Plain‐language summaries (PLS) are being heralded as a tool to improve communication of scientific research to lay audiences and time‐poor or nonspecialist healthcare professionals. However, this relies on PLS being intuitively located and accessible. This research investigated the “discoverability” of PLS in biomedical journals.

**Methods:**

The eLIFE list of journals/organizations that produce PLS was consulted on July 12, 2018, for biomedical journals (based on title). Internet research, primarily focusing on information provided by the journal websites, explored PLS terminology (what do the journals call PLS), requirements (what articles are PLS generated for, who writes/reviews them, and at what stage), and location and sharing mechanisms (where/how the PLS are made available, are they free to access, and are they visible on PubMed).

**Results:**

The methodology identified 10 journals from distinct publishers, plus *eLIFE* itself (N = 11). Impact factors ranged from 3.768 to 17.581. Nine different terms were used to describe PLS. Most of the journals (8/11) required PLS for at least all research articles. Authors were responsible for writing PLS in 9/11 cases. Seven journals required PLS on article submission; of the other four, one required PLS at revision and three on acceptance. The location/sharing mechanism for PLS varied: within articles, alongside articles (separate tab/link), and/or on separate platforms (eg, social media, dedicated website). PLS were freely available when they were published with articles; however, PLS were only included within conventional abstracts on PubMed for 2/11 journals.

**Conclusion:**

Across the few biomedical journals producing PLS, our research suggests there is wide variation in terminology, location, sharing mechanisms, and PubMed visibility. We advocate a more consistent approach to ensure that PLS have appropriate prominence and can be easily found by their intended audiences.

## 1 INTRODUCTION

Many patients and caregivers are eager to read about new treatments, be better informed regarding treatment choices, and get involved in making decisions about care.^1^, ^2^ In line with this, industry trends point to the public being increasingly embraced as valuable contributors to medical research,^4^ with the establishment of the Patients Included charters and the *BMJ* spearheading patient involvement in the journal editorial review process through their Patient and Public Partnership strategy.^5^, ^6^ Research shows that some journal editors feel that patient reviewers are beneficial to the peer‐review process,^7^ and that patient and public reviewers can assess the relevance and importance of research to patients and caregivers, and determine whether a treatment or intervention being studied is practicable and acceptable to patients.^8^ In addition, it has been reported that public involvement can enhance the design and implementation of clinical trials, for example, by increasing the quantity and quality of patient‐relevant priorities and outcomes.^9^ Enrolment, funding, and dissemination of trial data are other aspects of clinical trials that can reportedly be enhanced by public involvement.^9^

However, in order to support the moves toward patient and public involvement in medical research, scientific information needs to be more accessible, as the dense technical language of journal articles can be challenging for nonscientific audiences to understand.^10^ In recognition of the need to communicate research to lay audiences, some journals are starting to publish plain‐language summaries (PLS) as a component of full research articles. PLS help to make scientific research understandable and accessible by describing complex research using nontechnical language that can be easily understood. PLS are also important for transparency and enable greater reach, as a means to having an impact on the lives of patients, caregivers, and families affected by illness.^11^ As well as being of benefit to lay audiences, PLS are also likely to be used by time‐poor and nonspecialist healthcare professionals (HCPs) and researchers. Although experts may be used to reading technical scientific language, they may refer to PLS before deciding whether to read the full article, and PLS may help HCPs to quickly get an overview of large volumes of literature. With the increased uptake of online platforms and open‐access publishing, it is also likely that more patients will want to access specialized health‐related information.^12^ Therefore, PLS might in the future, if more ubiquitous, become vehicles that bring together HCPs, researchers, patients, caregivers, and the public.

A survey in 2018 has supported the value of PLS for both patients and physicians. After general internet searches (61%) and patient‐specific websites (57%), articles in scientific journals (47%) were the third most important online source of health‐related information for patients.^12^ Of the physicians who were surveyed, 60% indicated that they would use PLS, with 46% rating PLS as valuable. Interviews with patients highlighted that knowledge creates empowerment and that it is important that information is made accessible. Moreover, physician interviews indicated that PLS help generate dialog with patients and streamline communication whilst saving time, as patients are not solely dependent on them for information.^12^

Beyond journal article PLS, the value of summaries for lay audiences is also being evidenced by the emergence of clinical trial summaries. The European Union Trials Registration 536/2014 (Article 37) requires that a summary of clinical trial results understandable to a layperson be developed along with the clinical study report and be made publicly available on the EU database.^13^ In addition, in 2017, the United States Food and Drug Administration provided draft guidance on the provision of PLS for clinical trials.^14^ However, a publication in 2019 highlighted that at this time, less than 2% of all clinical trials completed or terminated within the past 3 years had returned PLS to study volunteers. This was below the forecast made 10 years previously when a pilot effort evaluated the feasibility and efficiency of a process for communicating summary results to patients. Nevertheless, a tightening of the regulatory environment and the growing appetite for PLS of clinical trial data is expected to push research sponsors to adopt PLS.^15^

While PLS are being heralded as a tool to improve communication of research to lay audiences and time‐poor HCPs, this will only be achieved if PLS are intuitively located and accessible. The aim of our research was to investigate how the “discoverability” of PLS is being handled by biomedical journals; the PLS for this article is presented in Box 1.

BOX 1 **Plain‐language summary for this article**

“Plain‐language summaries” (PLS) are short, easy‐to‐read summaries of medical research articles for lay readers. PLS are meant to be helpful to patients and the general public, but finding them can be hard, which will limit their use. We found that a range of names are used for PLS, which will make them hard to search for on the internet. Some names do not make it clear to lay readers, including patients, that the PLS are for them to use. Also, PLS are not available for all research articles, and when they are available, they are shared in different ways, such as on websites or via social media. Overall, we were pleased to find that PLS are free to read, but we recommend that ways of naming and sharing should be the same for all articles so that PLS are easier to find.

## 2 METHODS

A cross‐section of journals was identified based on the freely available eLIFE list of journals and other organizations that produce PLS.^16^ This list is compiled and maintained by eLIFE, a nonprofit organization that strives to improve all aspects of research communication in support of excellence in science. Users are invited to help keep the list up to date by contacting eLIFE with details of any suggested additions or updates.

The eLIFE list was consulted on July 12, 2018 for journals that we deemed relevant to the biomedical field (based on the journal titles). Internet research, primarily focusing on the information provided by the journal websites, explored PLS terminology (what do the journals call PLS), requirements (what articles are PLS generated for, who writes/reviews them, and at what stage), and location and sharing mechanisms (where/how the PLS are made available, are they free to access, and are they visible on PubMed). Internet research was conducted by one researcher (with findings populated in a data collection sheet), and subsequently checked by a second researcher. Separate follow‐up with specific journals was considered where key information was missing. The research was completed over the period July 12‐26, 2018.

## 3 RESULTS

### 3.1 Journals

The methodology initially identified 23 biomedical journals from the eLIFE list. These included one journal from each of the following publishers: BMJ Publishing (*Annals of the Rheumatic Diseases*), Canadian Science Publishing (*FACETS*), the Cochrane Library/Wiley (*Cochrane Database of Systematic Reviews*), eLIFE Sciences Publications (*eLIFE*), the National Academy of Sciences (*Proceedings of the National Academy of Sciences*), Sage Publishing (*Autism*), and Wiley (*Autism Research*). In addition, there were four publishers with multiple journals in the eLIFE list: PLOS (*PLOS Biology*, *PLOS Computational Biology*, *PLOS Genetics*, *PLOS Medicine*, *PLOS Neglected Tropical Diseases*, *PLOS Pathogens*), the National Institute for Health Research (*Efficacy and Mechanism Evaluation*, *Health Services and Delivery Research*, *Health Technology Assessment*, *Programme Grants for Applied Research*, *Public Health Research*), the American Chemical Society (*ACS Chemical Neuroscience*, *ACS Infectious Diseases*, *ACS Medicinal Chemistry Letters*), and Elsevier (*European Urology*, *Journal of Hepatology*). Journals from the same publishers were found to handle PLS in largely similar ways (see Appendix), and so in each case the journal with the highest impact factor was arbitrarily selected, to avoid potentially skewing the research: *PLOS Medicine*, *Health Technology Assessment*, *ACS Infectious Diseases*, and *European Urology*.

The above approach generated a final list of 10 journals from distinct publishers, plus *eLIFE* itself (N = 11). Impact factors ranged from 3.768 for *Autism Research* to 17.581 for *European Urology*. All journals were either open access (*eLIFE*, *FACETS*, *Health Technology Assessment*, *PLOS Medicine*), or offered optional open access (*ACS Infectious Diseases*, *Annals of the Rheumatic Diseases*, *Autism*, *Autism Research*, *Cochrane Database of Systematic Reviews*, *European Urology*, *Proceedings of the National Academy of Sciences*).

### 3.2 PLS terminology

Across the 11 journals, nine different terms were used to describe PLS: lay summaries (n = 3); lay abstracts (n = 2); plain‐language summaries (n = 2); patient summaries (n = 2); author summaries (n = 1); eLIFE digests (n = 1); plain English summaries (n = 1); scientific summaries for families with ASD (n = 1); and significance statements (n = 1). Moreover, two journals used more than one of these terms: *Annals of the Rheumatic Diseases* referred to PLS as both “lay summaries” (on the main journal website)^17^ and “patient summaries” (on the PLS archive website),^18^ and *Autism Research* had changed their term from “lay abstracts” to “scientific summaries for families with ASD,” and more recently to “lay summaries” (Table 1).

**Table 1:** Summary of journal approaches to handling PLS

	Terminology	Requirements			Location	Accessibility	PubMed visibility
*Journal*, Publisher	What were PLS called?	Were PLS required for all research articles?	Were PLS developed by authors?	When were PLS required?	Where were PLS housed?	Were PLS freely accessible?	Were PLS noted on PubMed?
*ACS Infectious Diseases*, American Chemical Society	Lay summaries	✓	✓	Submission	Not publicly available	Not publicly available	X
*Annals of the Rheumatic Diseases*, BMJ Publishing	Lay summaries/patient summaries	X^a^	X^b^	Acceptance	Archived on a separate website	✓	X
*Autism*, Sage Publishing	Lay abstracts	✓	✓	Acceptance	Supplemental material and via social media (Facebook)	✓	X
*Autism Research*, Wiley	Lay summaries (formerly “lay abstracts” and “scientific summaries for families with ASD”)	✓	✓	Submission	Within article and archived in separate section of journal website	✓	✓
*Cochrane Database of Systematic Reviews*, Cochrane Library/Wiley	Plain‐language summaries	✓	✓^c^	Submission	Within article and via separate navigation from article page	✓	X
*eLIFE*, eLIFE Sciences Publications	eLIFE digests	X^a^	X^b^	Acceptance	Within article and via social media (Medium)	✓	X
*European Urology*, Elsevier	Patient summaries	✓	✓	Submission	Within article and archived in separate section of journal website	✓	✓
*FACETS*, Canadian Science Publishing	Plain‐language summaries	X^d^	✓	Submission	Social media (Medium)	✓	X
*Health Technology Assessment*, National Institute for Health Research	Plain English summaries	✓	✓	Submission	Within article and via separate navigation from article page	✓	X
*PLOS Medicine*, PLOS	Author summaries	✓	✓	Revision	Within article and via separate navigation from article page	✓	X
*Proceedings of the National Academy of Sciences*, National Academy of Sciences	Significance statements	✓	✓	Submission	Within article and in separate section of issue table of contents	✓	X

Abbreviation: PLS, plain‐language summary/summaries.

a PLS only developed for articles selected by the editors.

b PLS developed by the editors based on author responses to questions.

c PLS may be developed by the authors themselves, or by the editorial team.

d Authors are encouraged to provide PLS—PLS only available for articles where they are volunteered by the authors.

### 3.3 PLS requirements

PLS were a requirement for all articles, or at least all research articles, in 8/11 journals. For the remaining journals, PLS were only developed for articles that were selected by the editors (n = 2) or authors were encouraged to provide PLS but these were not mandatory (n = 1) (Table 1).

For 9/11 journals, authors were responsible for writing PLS. *Health Technology Assessment* recommended that PLS development be supported by a Patient and Public Involvement representative, to ensure that language is appropriate for nonacademics. For the remaining journals, the journal editors developed PLS based on the authors' responses to questions (n = 1), or PLS could be developed by either the editorial team *or* the authors (n = 1) (Table 1).

Some journals provided details of additional review/editing steps for PLS: PLS in *Annals of the Rheumatic Diseases* were reported to be checked for accuracy and readability by expert rheumatologists and People with Arthritis and Rheumatism (PARE), which is the patient organization of the European League Against Rheumatism (EULAR); PLS in *Autism* were reported to be reviewed by a social media editor to ensure that they would be accessible to the target audience; PLS in *Cochrane Database of Systematic Reviews*, *eLIFE* or *PLOS Medicine* were reported to be edited by in‐house editorial team members.

Most journals required PLS upon submission (n = 7); however, for some journals, this was only necessary following article acceptance (n = 3) or during article revision (n = 1) (Table 1).

The required format for PLS was not a focus of this research, but there appeared to be broad variation in the level of instruction provided and where this could be found. For example, where word limits were specified, these ranged from 60‐80 words (*Autism Research*) to 250‐500 words (*FACETS*); meanwhile, the instructions to authors for *Annals of the Rheumatic Diseases* did not include reference to PLS, but typical published PLS appeared to be approximately 750‐850 words. Some journals requested that PLS be targeted to an audience with some scientific knowledge (eg, the level of a biochemistry undergraduate [*ACS Infectious Diseases*] or “an undergraduate‐educated scientist outside [of the] field” [*Proceedings of the National Academy of Sciences*]), while others requested targeting to a lay audience (eg, the level of “interested persons without a scientific background” [*Autism Research*] or a “non‐medical audience” [*European Urology*]). The few journals that offered relatively detailed guidance included *eLIFE* and *Health Technology Assessment*.^19^, ^20^ Regarding the *Cochrane Database of Systematic Reviews*, it was difficult to locate clear instructions around PLS, perhaps because author teams work closely with Cochrane Review Groups who manage the editorial process; nonetheless, it was determined that authors are required to work to Cochrane‐approved standards outlined in Plain Language Expectations for Authors of Cochrane Summaries (PLEACS), established in 2013.^21^

### 3.4 PLS locations/sharing mechanisms

The locations/sharing mechanisms of PLS varied between journals, with some journals including them within articles, alongside articles (via a separate tab/link), and/or on separate platforms such as social media or via a dedicated website (Table 1). For one journal, *ACS Infectious Diseases*, PLS did not appear to be publicly available, and when contacted by email, the journal indicated that these PLS were only available to the press. Where PLS were published with articles (n = 10/11), they were still freely accessible even when the main article was behind a paywall.

PLS were only included with conventional abstracts on PubMed for 2/11 journals: *Autism Research* and *European Urology*. In both cases, authors were required to submit short PLS as part of the conventional abstract, and so the PLS appeared within the abstracts on PubMed, rather than as separate entities. The *Autism Research* website highlighted that this approach was intended to ensure that PLS are available to any reader, free of charge.

## 4 DISCUSSION

Our research suggests that there are few biomedical journals inviting PLS for publication, which is in line with previous observations.^22^ Moreover, amongst the few biomedical journals that were identified, we found wide variation in PLS terminology, requirements, and locations/sharing mechanisms.

Ultimately, our methodology relied on the eLIFE list of journals producing PLS. However, we had previously found that the variation in PLS terminology made it challenging to define a systematic and robust sampling methodology for identifying a representative cross‐section of biomedical journals that publish PLS. As “PLS” is not a term used universally across journals, there were no logical search criteria that could be inputted into databases of biomedical literature (eg, PubMed), without potentially introducing bias by focusing on the terms that were already known to us. If standard internet search engines were used, this would have been further compounded by the fact that the term “PLS” is not exclusive to the biomedical field. By extension, we envisage that this terminology issue could also hamper the efforts of target audiences to find PLS as well.

We also found that PLS terminology did not necessarily clearly reflect the target audience. For example, lay audiences who come across “lay abstracts/summaries” or “patient summaries” may suspect these could be relevant for them to read, but we suggest they might overlook “author summaries” or “significance statements.” In our experience, some journals require authors to develop bulleted “key points” or “significance statements” to provide a bite‐sized overview of articles for time‐poor HCPs. These are not typically developed with lay audiences in mind but, given the evolving journal article PLS landscape, sometimes appear to be categorized under the “PLS” umbrella, which is confusing. So beyond the need for more consistent PLS terminology, we also believe that there needs to be better distinction between, and clearer naming of, summaries intended for broad use, including by lay audiences, vs those bite‐sized overviews specifically designed for time‐poor or nonspecialist HCPs and researchers. The two serve different purposes and the medical communications industry needs to help lay audiences find content that could be helpful to them, by naming it consistently while differentiating it clearly from content directed solely at specialist HCPs and researchers. PLS for broad use need not only to be pitched appropriately but should also consider points that are relevant from the patient perspective (without deviating from the content in the published article); this will be another differentiator vs the bite‐sized overviews directed at HCPs and researchers.

PLS requirements varied between journals in terms of which articles PLS are developed for, who writes the PLS, what review processes are involved, and when the PLS should be submitted. To some extent this reflects general inconsistency in approaches, but may not directly impact PLS discoverability. Having said that, if there is inconsistency in which articles PLS are developed for, then some readers may be quick to assume that there is no PLS rather than taking the time to determine if a PLS is just difficult to find. While it was beyond the scope of this research to consider PLS formats, there was evidence that formats also varied widely between journals, which is in line with the findings of others.^22^ We would fully support further investigation of PLS formats, including within‐journal variation (given the limited guidance offered by some journals), as well as the quality and appropriateness of PLS content.

Among the small sample of journals producing PLS, all but one made PLS freely accessible. In most cases, PLS were published as part of the full article, but some were shared via social media, or within scientific abstracts on PubMed. The inconsistency in approaches made it difficult to locate some PLS. For example, information about PLS was not prominent on the main website of *Annals of the Rheumatic Diseases*, and it took some time to locate where PLS were housed on a separate website. Had the eLIFE list not identified this as a journal that produced PLS, then, based on the main journal website alone, we may well have assumed that no PLS were available. This highlights how there is no one‐size‐fits‐all approach for locating PLS, even via a journal website, and how easy it could be for PLS to remain undiscovered by their target audiences.

Maximizing the potential for reaching intended audiences should be a key consideration when thinking about the appropriateness of sharing mechanisms and each approach has benefits in different scenarios. Lay audiences seeking out original research articles will value a PLS integral to the article as an access point to further reading. Meanwhile, being able to access and collate PLS for specific disease areas from searchable archives will enable readers to build an evolving picture of published research and to forego complex scientific articles written for experts; sharing PLS via social media is likely to push the latest research to interested audiences quickly. We also suggest that publishing PLS within journal articles alongside the other traditional article sections (such as the abstract) sends an important signal about valuing communication with wider audiences and being genuine, rather than tokenistic, about the desire to involve patients in all aspects of medical research. For the same reason, it is interesting that we found some PLS integral to conventional abstracts on PubMed. The growing interest in PLS is challenging journal publishers and PubMed alike to respond to evolving target audiences and their changing needs. Databases are already available to host PLS for journal articles where the publisher does not offer a PLS option. For example, *BMJ* is partnering with Kudos (www.growkudos.com) to offer a platform for the development and hosting of PLS for all articles that is distinct from, but, importantly, links to, published articles.

Overall, our research begs the question of why, in a time when the value of the patient voice is being embraced, and time‐poor and nonspecialist HCPs need more “snackable” content, PLS are not being offered by more journals—or even becoming a mandated part of article requirements in journal instructions to authors. Obstacles to PLS uptake could include the possibility that, where PLS focus more on points of relevance to patients, they may appear to have a different balance of content compared with the article's abstract, which could prompt criticism or even perceptions of direct‐to‐consumer promotion. Perceived duplicate publication is another challenge, but requiring that PLS do not generalize findings, are peer‐reviewed alongside all other elements of an article, and are published within that article or on a linked platform referencing the original citation, will make these issues surmountable.^23^

A key limitation of this research is the reliance on the eLIFE list of journals that produce PLS. Given the above challenges relating to terminology, use of the eLIFE list was deemed the most practical methodology for identifying an unbiased sample for exploring PLS accessibility. However, we had no assurance that this list was complete, and suspect that some relevant journals will not have been captured. For example, we were aware that Adis journals support publication of PLS, but these were not included in the eLIFE list (perhaps because the Adis journals are among a growing number of journals that do not mandate PLS but will publish PLS when requested by authors).^24^ Since conducting this research in the summer of 2018, other possible methodologies have come to light, as we have become aware of the PubMed feature to filter for articles that include information for patients (via the search term “hassummaryforpatientsin”).^25^ Moreover, in February 2019, PubMed announced that when PLS are supplied by the publisher, these will appear below the abstract^26^; they also introduced the “plain‐language‐summary” meta‐tag, although so far uptake appears to be low amongst journals publishing PLS.^27^ If such functionality was widely recognized and applied, it could be extremely useful for looking at the frequency of PLS for biomedical articles in future research. Another, more recent, development that could aid identification of journals publishing PLS is the introduction of “Plain‐Language Summaries” as a searchable metric in the journal database of the widely used publication management tool Datavision (Envision Pharma Group). This highlights how the opportunity to include PLS is increasingly becoming a metric of interest to guide journal selection.

A further limitation is that a systematic approach to the journal research was not possible due to the wide variation in how journal websites are structured, how much information about PLS is made available, and how prominent that information is. Although researchers took a thorough approach, it is possible that relevant information could have been overlooked, due to the issues of inconsistency that the research itself has highlighted.

**Figure 1:**
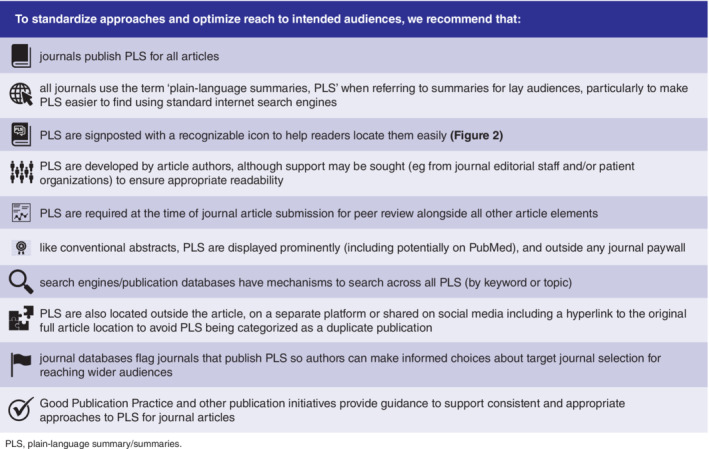
Our recommendations for PLS for journal articles. PLS, plain‐language summary/summaries

**Figure 2:**
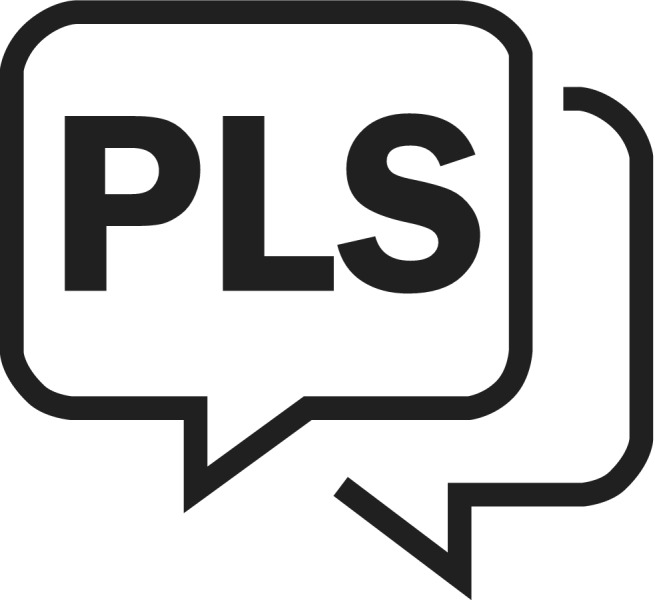
Our recommended icon to identify PLS in journal articles. PLS, plain‐language summary/summaries

Based on our research, and our knowledge of the field, a summary of our own recommendations for PLS is presented in Figure 1. To increase the reach of biomedical research to lay audiences, we advocate that journals mandate the requirement for PLS for all articles. The 11 journals studied in this research referred to PLS using nine different descriptors between them, so another key recommendation would be that all journals refer to summaries for lay audiences by the consistent *plain‐language summaries (PLS)* descriptor to make them easier to find using standard internet search engines, and so audiences know that they are looking for the same thing whatever the journal. Using a standard, instantly recognizable icon, such as that in Figure 2, to signpost PLS within an article would also help readers locate them easily. PLS should be developed as part of the full scientific article, by the authors (with professional writing support to ensure appropriate readability)^3^ and with patients/lay people where possible, and be required at journal article submission so PLS can be peer‐reviewed alongside all other elements of the article. They should be published outside any article paywall and be positioned or signposted to have equal prominence with other key parts of the article. Where PLS are located on an associated platform or website, they must always include a link to the original full article to avoid PLS being categorized as a duplicate publication. It would be helpful if other databases follow Datavision's lead in flagging journals that publish PLS so authors can make informed choices about target journal selection for reaching wider audiences. Incorporation of guidance to support consistent and appropriate approaches to PLS in Good Publication Practice (GPP) guidelines^28^ would also improve education in this area and we understand this will be part of the GPP4 update due in 2021.

## 5 CONCLUSIONS

Patients and the public can provide critical added value to research. Some journals are starting to recognize this through PLS. Our research found that the terminology, location, sharing mechanisms, and visibility on PubMed of PLS differed between the journals studied. We suggest that a more consistent approach to PLS would enable biomedical research to be communicated to wider audiences. Through achieving this, PLS could become a tool that brings together HCPs, patients and the public.

## FUNDING

This research was funded by McCann Health Medical Communications, the current or former employer of all authors. A draft version of the article was reviewed by members of the McCann Health Medical Communications research and peer review team.

## CONFLICT OF INTEREST

Hannah FitzGibbon is an employee of CMC Affinity and CMC Connect, McCann Health Medical Communications. Karen King and Mary Gaskarth are employees of CMC Affinity, McCann Health Medical Communications. Claudia Piano is a former employee of CMC Affinity and CMC Connect, McCann Health Medical Communications. Carol Wilk is a former employee of CMC Affinity, McCann Health Medical Communications, and a current employee of Peloton Advantage.

## AUTHOR CONTRIBUTIONS

Conceptualization: Hannah FitzGibbon, the corresponding author, confirms that she had full access to all of the data in the study and takes responsibility for the integrity of the data and the accuracy of the data analysis.

Investigation: Carol Wilk

Methodology: Hannah FitzGibbon, Karen King, Claudia Piano, Carol Wilk, Mary Gaskarth

Writing – original draft preparation: Hannah FitzGibbon, Karen King, Carol Wilk, Mary Gaskarth

Writing – review and editing: Hannah FitzGibbon, Karen King, Claudia Piano, Carol Wilk, Mary Gaskarth.

All authors have read and approved the final version of the manuscript.

Hannah FitzGibbon, the corresponding author, confirms that she had full access to all of the data in the study and takes responsibility for the integrity of the data and the accuracy of the data analysis.

## TRANSPARENCY STATEMENT

As corresponding author, Hannah FitzGibbon confirms that the manuscript is an honest, accurate and transparent account of the study being reported; that no important aspects of the study have been omitted; and that any discrepancies from the study as planned have been explained.

## Data Availability

Some of these results have been presented as posters at the International Society for Medical Publication Professionals (ISMPP) 2019 European and 15th Annual Meeting. The datasets used and/or analyzed during the current research are available from the corresponding author on reasonable request.

## 1

While the research only included the highest impact factor journals by each publisher, other journals by the same publishers handled PLS in largely similar ways according to the parameters researched and reported here.

The six PLOS journals were similar, except that two required PLS to be submitted at journal article revision (*PLOS Biology*, *PLOS Medicine*) rather than at submission (*PLOS Computational Biology*, *PLOS Genetics*, *PLOS Neglected Tropical Diseases*, *PLOS Pathogens*).

The five National Institute for Health Research (NIHR) journals were similar, except that one did not include a link out to the PLS from PubMed (*Health Technology Assessment*), while the others did (*Efficacy and Mechanism Evaluation*, *Health Services and Delivery Research*, *Programme Grants for Applied Research*, *Public Health Research*).

All three journals by the American Chemical Society (*ACS Chemical Neuroscience*, *ACS Infectious Diseases*, *ACS Medicinal Chemistry Letters*) had identical approaches according to the reported parameters.

The two Elsevier journals were similar, except that one referred to “lay summaries” (*Journal of Hepatology*) and the other “patient summaries” (*European Urology*), and one required PLS to be submitted at journal article revision (*Journal of Hepatology*) rather than at submission (*European Urology*).
